# Inhibition of Tryptophan Catabolism Is Associated With Neuroprotection During *Zika* Virus Infection

**DOI:** 10.3389/fimmu.2021.702048

**Published:** 2021-07-15

**Authors:** Fernanda Martins Marim, Danielle Cunha Teixeira, Celso Martins Queiroz-Junior, Bruno Vinicius Santos Valiate, Jose Carlos Alves-Filho, Thiago Mattar Cunha, Robert Dantzer, Mauro Martins Teixeira, Antonio Lucio Teixeira, Vivian Vasconcelos Costa

**Affiliations:** ^1^ Department of Biochemistry and Immunology, Institute of Biological Sciences, Universidade Federal de Minas Gerais, Belo Horizonte, Brazil; ^2^ Research Group in Arboviral Diseases, Institute of Biological Sciences, Universidade Federal de Minas Gerais, Belo Horizonte, Brazil; ^3^ Center for Drug Research and Development of Pharmaceuticals, Institute of Biological Sciences, Universidade Federal de Minas Gerais, Belo Horizonte, Brazil; ^4^ Departament of Morphology, Institute of Biological Sciences, Universidade Federal de Minas Gerais, Belo Horizonte, Brazil; ^5^ Center for Research in Inflammatory Diseases (CRID), Department of Pharmacology, Ribeirao Preto Medical School, Universidade de São Paulo, Ribeirão Preto, Brazil; ^6^ Department of Symptom Research, The University of Texas MD Anderson Cancer Center, Houston, TX, United States; ^7^ Department of Psychiatry and Behavioral Sciences, McGovern Medical Houston, The University of Texas Health Science Center at Houston, Houston, TX, United States

**Keywords:** *Zika virus*, IDO-1, neuroinflammation, neuronal death, microgliosis

## Abstract

*Zika virus* (ZIKV) is an arbovirus belonging to *Flaviviridae* family that emerged as a global health threat due to its association with microcephaly and other severe neurological complications, including Guillain-Barré Syndrome (GBS) and Congenital Zika Syndrome (CZS). ZIKV disease has been linked to neuroinflammation and neuronal cell death. Neurodegenerative processes may be exacerbated by metabolites produced by the kynurenine pathway, an important pathway for the degradation of tryptophan, which induces neuronal dysfunction due to enhanced excitotoxicity. Here, we exploited the hypothesis that ZIKV-induced neurodegeneration can be rescued by blocking a target enzyme of the kynurenine pathway, the Indoleamine 2,3-dioxygenase (IDO-1). RT-PCR analysis showed increased levels of IDO-1 RNA expression in undifferentiated primary neurons isolated from wild type (WT) mice infected by ZIKV *ex vivo*, as well as in the brain of ZIKV-infected A129 mice. Pharmacological inhibition of IDO-1 enzyme with 1-methyl-D-tryptophan (1-MT), in both *in vitro* and *in vivo* systems, led to significant reduction of ZIKV-induced neuronal death without interfering with the ability of ZIKV to replicate in those cells. Furthermore, *in vivo* analyses using both genetically modified mice (IDO^-/-^ mice) and A129 mice treated with 1-MT resulted in reduced microgliosis, astrogliosis and Caspase-3 positive cells in the brain of ZIKV-infected A129 mice. Interestingly, increased levels of CCL5 and CXCL-1 chemokines were found in the brain of 1-MT treated-mice. Together, our data indicate that IDO-1 blockade provides a neuroprotective effect against ZIKV-induced neurodegeneration, and this is amenable to inhibition by pharmacological treatment.

## Introduction

ZIKV is an arbovirus belonging to the family *Flaviviridae*, genus *Flavivirus*, composed of positive sense, single-stranded RNA. ZIKV was first isolated from a rhesus monkey in 1947 in the Zika forest (Uganda) and humans’ infection was reported in Nigeria in 1954 (1). Epidemiological studies suggest that *Zika virus* had a wide geographical distribution, being introduced in Brazil between 2013 and 2015, causing a major epidemic (2) when there was an increase in cases of microcephaly and the incidence of Guillain-Barré syndrome (GBS) (3). ZIKV transmission occurs mainly by culicids of the genus *Aedes* (4), however, other transmission routes such as blood transfusion, sexual transmission and maternal-fetal transmission have already been demonstrated (5).

In humans, the disease caused by ZIKV manifests in about 20% of cases and is characterized by mild clinical symptoms such as fever, headache, myalgia, rash, conjunctivitis and joint pain (6). However, fetal brain samples from infected mothers have shown that ZIKV is able to break through biological placental barriers to infect developing neural cells inducing neuroinflammation, neuronal death, and neurodegeneration, therefore, leading to microcephaly and congenital Zika syndrome, both of which eventually manifest as fetal brain abnormalities (7–11).

The mechanism by which ZIKV exerts these neurological effects has been related to induction of neuronal excitotoxicity, a pathological process mediated by excessive glutamatergic activity (12), infection, activation, and apoptosis of neural progenitor cells, mature neurons, and glial cells with concomitant inflammation (13). Cell death and neurodegenerative processes can be exacerbated by metabolites produced by the kynurenine pathway (KP), an important pathway of degradation of tryptophan, at least in part due to excitotoxicity. Kynurenine pathway is induced by the activation of Indoleamine-2,3-dioxygenase (IDO-1) enzyme in the presence of proinflammatory cytokines, generating several neuroprotective and neurotoxic metabolites (14, 15).

IDO-1 is found in macrophages, monocytes, microglia, astrocytes, and neurons (16, 17) and is considered the major enzyme involved in tryptophan degradation (18). Imbalances in levels of KP metabolites have been associated with neurodegenerative disorders including Huntington’s, Alzheimer’s, and Parkinson’s diseases and other brain disorders as well as several cancers (19). IDO-1 has been linked to neurodegenerative diseases, and have been studied as a possible therapeutic target (19–23).

1-methyl-D-tryptophan (1-MT) is a methylated tryptophan, acting as a competitive IDO-1 inhibitor (24). Recent studies have shown its ability to improve responses to many anticancer therapies and to boost immunity in infectious diseases (24).

In this study we explored the hypothesis that ZIKV-induced neurodegeneration can be rescued by blocking the enzyme IDO-1 that catalyzes the rate-limiting step in KP.

## Material and Methods

### Animals and Ethics

This study was carried out in accordance with the recommendations of the Brazilian Government (law 11794/2008a) and approved by the Committee on Animal Ethics of Universidade Federal de Minas Gerais (UFMG) (protocol no.106/2020 CEUA/UFMG). All mice were 5 to 8 weeks old and were kept under specific-pathogen-free conditions at 23°C on a 12-h light/12-h dark cycle with food and water provided *ad libitum*. Experiments were conducted using wild-type C57BL/6 mice, type I interferon receptor deficient mice (A129) on SV129/Ev background, and deficient mice in the enzyme indoleamine 2,3-dioxygenase (IDO-1^-/-^) on C57BL/6 background at the Immunopharmacology Laboratory at ICB-UFMG. C57BL/6 mice were purchased from Biotério Central of UFMG, A129 mice were originally purchased from B&K Universal Limited (United Kingdom) and IDO-1^-/-^ were kindly provided by Universidade de São Paulo (USP).

### Mouse Experiments

Mice were inoculated with ZIKV by intravenous (I.V) route (tail vein) with 4x10^3^ PFU of ZIKV in a volume of 200 μl PBS or by intracranial (i.c) route with 1x 10^6^ PFU of ZIKV in a volume of 20 μl PBS. In some experiments, mice were treated orally with 10 mg/animal of the Indoleamine 2,3-dioxygenase enzyme inhibitor (1-MT), 1 hour after infection and every 24 hours. Clinical symptoms were monitored daily and severely ill mice with a ≥ 20% weight decrease were euthanized. On day 5 after infection (the peak of ZIKV infection), the animals were euthanized to obtain spleen, brain, optic nerve and eye. For primary culture experiments (neurons), brains from mouse embryos on the fifteenth day of the embryonic period (E15) were collected to obtain neuronal cultures.

### Virus

A low-passage-number clinical isolate of ZIKV (HS-2015-BA-01), isolated from a viremic patient with symptomatic infection in Bahia State, Brazil, in 2015, was used. The complete genome of the virus is available at GenBank under the accession no. KX520666. Virus stocks were propagated in C6/36 *Aedes albopictus* cells and were titrated in CCL-81 Vero cells as described previously (25).

### Intraocular Pressure Measurement (IOP)

The IOP was measured on days 0, 3 and 5 after ZIKV infection using an applanation tonometer (Tono-Pen Vet - Reichert Technologies, NY, USA), as previously described (26–28).

### Virus Titration

The viral load on mouse tissues (brain, eye, optic nerve and spleen) and in the supernatant of cell culture samples was determined by plaque assay in CCL-81 Vero cells, as previously described (25). Plaque counts were computed as p.f.u. per gram of tissue mass or milliliter of supernatant.

### Evaluation of Inflammatory Markers

The brain tissue was homogenized in a buffer containing protease inhibitors (100 mg of tissue per 1 mL of extraction solution; 0.4 mol/L NaCl, 0.05% Tween 20, 0.5% BSA, 0.1 mmol/L phenylmethyl sulfonil fluoride, 0.1 mmol/L benzethonium chloride, 10 mmol/L EDTA, and 20 KI aprotinin). The brain homogenate was centrifuged at 3000 × *g *for 10 min at 4°C, and the supernatant was collected to determine the concentration of cytokines by ELISA. The cytokines (TNF and IL-1β) and chemokines (CCL5 and CXCL1) levels in the brain of mice were measured using DuoSet ELISA kits antibodies (R&D Systems) in accordance with the manufacturer’s instructions. Neutrophil accumulation in mouse brains was also indirectly measured by determining the level of myeloperoxidase activity, according to a previous study (29).

### Brains Sections and Staining

For histopathological scoring, brain samples were stained with hematoxylin and eosin (H&E) and the analyses was performed in cerebral cortex and hippocampus sections in a blinded manner according to previous studies (29, 30). Briefly, each brain region was graded on a four-point scale: 0, no tissue damage; 1, minimal tissue damage and/or mild inflammation; 2, mild tissue damage and/or moderate inflammation; 3, severe tissue damage and high inflammation; 4, necrosis with loss of tissue elements and presence of cellular debris. Meningeal inflammation was also graded on a four-point scale: 0, no inflammation; and points between 1 and 4 were assigned when there were one to four layers of cellular inflammation, respectively. The final score was calculated as a sum of cerebral cortex added to the score obtained from the meningeal inflammation analysis, totalizing a maximum of 8 points. For immunostaining analyses, brain samples were processed and sections from hippocampus, striatum, and prefrontal and motor cortex of mice were stained for microglia (IBA1 Polyclonal Antibody – Invitrogen), astrocytes (S100-β polyclonal antibody – Abcam) and apoptosis (Caspase-3 Monoclonal Antibody – Invitrogen) according to manufacturer’s instructions (Vector Elite kit - Vector Laboratories) as described in as previously described (31). Image acquisition and analysis were performed using an Olympus BX 41 microscope (Olympus). The images presented in the article are representative of one of those experiments.

### Primary Neurons Cell Cultures

Neuronal cultures were prepared from the cortex and striatal regions of E15 of C57BL/6 wild-type mouse embryo brains. After dissection, the brain tissue was submitted to trypsin digestion followed by cell dissociation using a fire-polished Pasteur pipette. Neuronal cells were plated onto poly-L-ornithine-coated dishes in neurobasal medium supplemented with N2 and B27 supplements (Gibco), 2 mM GlutaMAX (Gibco) and 50 g/ml penicillin/streptomycin (Sigma), incubated at 37°C and 5% CO_2_ for 5 days. Infection was performed with ZIKV (MOI of 0.1), followed by an adsorption period of 1 h. Wells were washed with incomplete medium and each well was replaced by a final volume of complete neurobasal medium. In some experiments, neuronal cultures were treated with 3, 10, 30 or 100 µM of the Indoleamine 2,3-dioxygenase enzyme inhibitor every 24h. Beside the kinetic experiments, all experiments evaluating the effects of 1-MT on primary neurons were performed after 48 h of ZIKV infection. At the time point, the supernatant was collected for quantification of viral load while the cells adhered to the plate were used in the cell death assay.

### Neuronal Cell Viability

Neuronal cell death was assessed by LIVE/DEAD Cell Viability Assays after 48 hours of ZIKV infection, as previously described (27). Briefly, the neuronal cell culture was stained with 2 µM calcein acetoxymethyl ester (AM) and 2 µM ethidium homodimer -1. After 10 minutes, the neuronal culture was immediately visualized by a FLoid Cell Imaging Station fluorescence microscope (Thermo Fisher Scientific). Images were captured and the fractions of live (green, calcein AM) and dead (red, ethidium homodimer -1) cells in the same field of view were evaluated. Cell viability was analyzed, and the quantification was performed using the ImageJ software. Data are reported as the percentage of dead cells out of the total number of cells.

### Real-Time RT-PCR

RNA was isolated using TRIzol™ reagent in accordance with the manufacturer’s instructions (Invitrogen). RNA was resuspended in 30 μL of nuclease-free water, and its concentration was analyzed by Nanodrop spectrophotometer. cDNAs were prepared from 2 μg of total RNA extracted in a 10 μL final reverse transcription reaction. RT-qPCR was performed from 10x diluted cDNA using SYBR Green PCR Master Mix (Applied Biosystems). Real-time was performed in a 7500 Fast Real-Time PCR System (Applied Biosystems). The appropriate primers were used to amplify a specific fragment corresponding to specific gene targets as follows: GAPDH F: 5’-ACG GCC GCA TCT TCT TGT GCA- 3’; GAPDH R: 5’ -CGC CCA AAT CCG TTC ACA CCG A- 3; IDO-1 F: 5’-TCA AAG CAA TCC CCA CTG TAT CC- 3’; IDO-1 R: 5’ -TCC ACA AAG TCA CGC ATC CTC- 3’. All data are presented as relative expression units after normalization to GAPDH gene, and measurements were conducted in duplicate.

### Statistical Analysis

Results were expressed as Mean ± SEM for the number of independent experiments. GraphPad Prism (GraphPad Software, Inc) was used to analyze data using different-tests as appropriate and *P < 0.05*, *P < 0.01*, *P < 0.001* and *P < 0.0001* indicate the levels of statistical significance. Additional information is indicated in *Figure Legends*.

## Results

### IDO-1 Expression Is Enhanced Upon ZIKV Infection *In Vitro* and *In Vivo*

**Figure 1:**
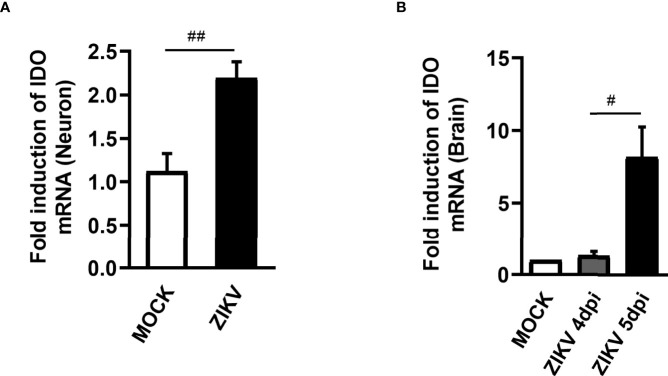
IDO-1 expression induced by ZIKV infection. **(A)** Primary culture of cortical-striatal neurons from C57BL/6 mice on D5 were infected with ZIKV (MOI 0.1) and after 48 hours the cells were harvested. **(B)** Type I interferon receptor deficient mice (A129) were inoculated (i.v) with 4x10^3^ PFU/200 μL of ZIKV. At day 4 and 5 of infection, the brain was harvested. RNA extraction and subsequent Real time PCR analysis of IDO-1 expression in the brain and neuronal primary culture was done. Results are expressed as mean ± SEM and are representative of two independent experiments. Statistically significant differences were assessed by Mann Whitney test. (#) for *P ˂ 0.05* and (##) for *P ˂ 0.01*.

The KP is segregated into two distinct branches triggering neurotoxic and neuroprotective metabolites. The first step of KP converts the essential amino acid L-tryptophan to N-formylkynurenine and is regulated by rate-limiting enzymes, being the Indoleamine 2,3-dioxygenase 1 (IDO-1) the main enzyme contributing to the production of kynurenines during inflammatory conditions (32). Therefore, differential mRNA expression of IDO-1 was investigated in non-infected and ZIKV-infected primary neuron cultures from cortex and striatal regions obtained from wild type (WT) C57BL/6 mice embryo brains. After 48 hours of ZIKV infection, there was an increase in the levels of IDO-1 when compared to Mock control in primary neuron cultures (**Figure 1A**). Additionally, increased levels of IDO-1 expression were also seen in the brain of A129 mice infected with ZIKV, 5 days upon infection, peak of disease manifestation in these mice, when compared to Mock littermates (**Figure 1B**). These results clearly demonstrate that ZIKV infection induced increased expression of IDO-1 enzyme *in vitro* and *in vivo*.

### 1-MT Inhibitor Reduces Neuronal Death in ZIKV Primary Neurons

**Figure 2:**
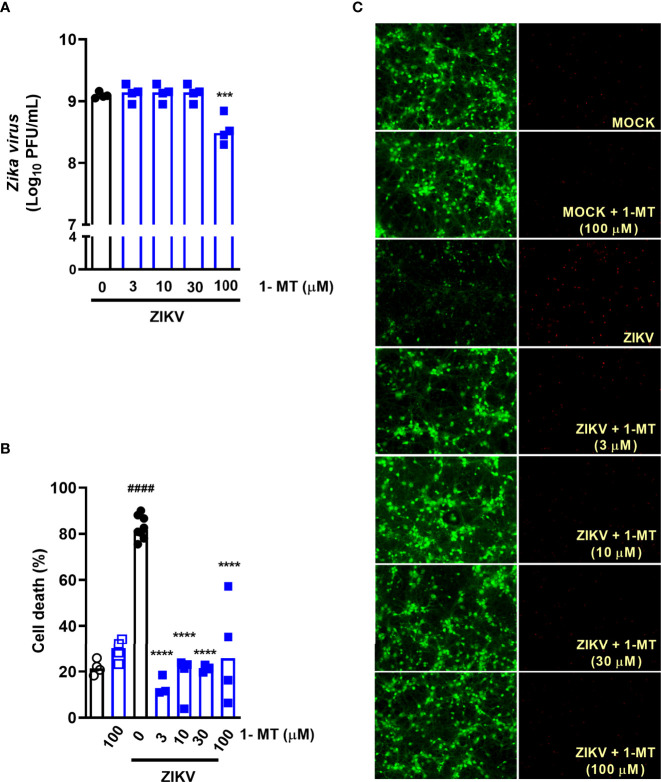
IDO-1 inhibition in primary undifferentiated neurons infected by ZIKV. Primary culture of cortical-striatal neurons on D5. Neuronal culture from C57BL/6 mice were infected with ZIKV (MOI 0.1) and treated with 1-MT inhibitor at concentrations of 3; 10; 30 and 100 µM. After 48 hours, **(A)** the supernatant was collected to assess viral load, **(B)** cell death was assessed using the LIVE/DEAD Cell Viability Assay. **(C)** Representative images of infected and uninfected neurons stained with calcein AM (green indicates live cells) and ethidium homodimer (red indicates dead cells). All results are expressed as median and are representative of at least two independent experiments. Statistically significant differences were assessed by One-way ANOVA plus Holm-Sidak’s or Tukey’s comparisons test. (####) for *P < 0,0001* compared to the MOCK group; (***) for *P < 0,001* and (****) for *P < 0,0001* compared to the ZIKV group.

Subsequently, we evaluated the effect of blockade of IDO-1 enzyme *in vitro.* Previous studies by our group have shown that undifferentiated neuronal cultures are highly susceptible to ZIKV infection, inducing neuronal death (27, 33). Then, we tested whether the IDO-I inhibitor, 1-methyl-D-tryptophan (1-MT) would reduce ZIKV-induced neuronal cell death. For that, primary neuronal cultures obtained from the corticostriatal region of C57BL/6 embryo brains were infected with ZIKV (MOI 0.1) and treated with 1-MT at concentrations of 3; 10; 30 and 100 µM. After 48 hours following infection, the supernatant was collected to evaluate viral load by plaque assay and cells were subjected to cell viability assays. ZIKV neuronal infection resulted in 10^9^ PFU/mL in the culture supernatants, and *in vitro* IDO-1 inhibition with 1-MT did not interfere with ability of the virus to replicate in these cells. The highest 1-MT concentration triggered some reduction of the viral load (**Figure 2A**). Nevertheless, 1-MT treatment induced a significant reduction of neuronal cell death, in all evaluated concentrations (**Figures 2B, C**). At the concentration of 100 µM 1-MT, cytotoxicity was detected in infected cells, but without reaching statistical difference.

### IDO-1 Inhibition Did Not Prevent ZIKV-Induced Clinical Manifestations as Well as Virus Replication in A129 Mice

**Figure 3:**
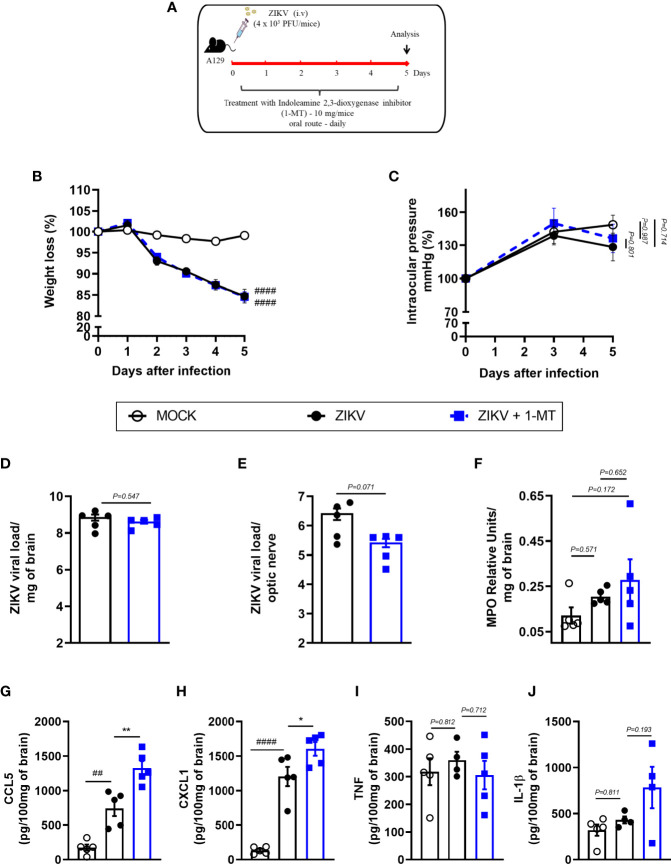
Clinical signs and inflammatory parameters in A129 mice infected by ZIKV treated or not with 1-MT inhibitor. **(A)** Experimental schematic representation of experimental strategy. A129 were inoculated (i.v) with 4x10^3^ PFU/200μL of ZIKV and treated daily with 1-MT. **(B)** The body weight was evaluated daily and **(C)** the intraocular pressure was analyzed on days 0, 3 and 5. At day 5 of infection, **(D)** the brain and **(E)** optical nerve was harvested for plaque assay analysis. **(F)** Measurement of myeloperoxidase activity (neutrophil accumulation) and **(G–J)** chemokine/cytokine production was assessed in mouse brain of animals. All results are expressed as mean and error bar indicate the standard error (SEM) and are representative of at least two independent experiments. Statistically significant differences were assessed by Two-way ANOVA plus Tukey’s comparisons test **(A, B)**, Mann Whitney test **(D, E)** and One-way ANOVA **(F–J)** plus Tukey’s comparisons test. (##) for *P < 0,01* and (####) for *P < 0,0001* when compared to the MOCK group. (*) for *P < 0,05* and (**) for *P < 0,001* compared to the ZIKV group.

Further, we investigated the effects of IDO-1 inhibition during ZIKV infection *in vivo*. For that, A129 mice were inoculated (i.v) with 4 x 10^3^ PFU of ZIKV and, after 1 hour, they were treated with 1-MT and every 24 hours until euthanasia (**Figure 3A**). Five days after infection, peak of disease manifestation in this model, spleen, brain, optic nerve and eye of the animals were collected. In accordance with previous studies from our group (27), there was significant body weight loss in the ZIKV group when compared to Mock control. Similarly, infected mice treated with 1-MT lost as much weight as the ZIKV group (**Figure 3B**). Ophthalmic alterations induced by ZIKV infection were also evaluated through the measurement of intraocular pressure (IOP), but no significant differences were detected between infected groups, treated or not with 1-MT (**Figure 3C**). Moreover, 1-MT treatment of ZIKV-infected mice did not interfere with viral loads detected in the brain (**Figure 3D**), optic nerve (**Figure 3E**), eye (**Figure S1A**) or spleen (**Figure S1B**). No significant differences were detected in neutrophils recruitment to the brain (**Figure 3F**). Although 1-MT did not prevent viral replication, it induced larger production of the chemokines CCL5 (**Figure 3G**) and CXCL1 (**Figure 3H**) in the brain of infected mice when compared to non-treated ZIKV group, but there was no difference in TNF (**Figure 3I**) and IL -1β (**Figure 3J**) levels. Overall, the results show that IDO-1 inhibition was not able to reduce clinical manifestations induced by ZIKV-infection, as well as virus replication in A129 mice.

### IDO-1 Inhibition Prevents Microgliosis, Astroglioses and Caspase-3 Expression of Cells in the Brain of ZIKV Infected Mice

**Figure 4:**
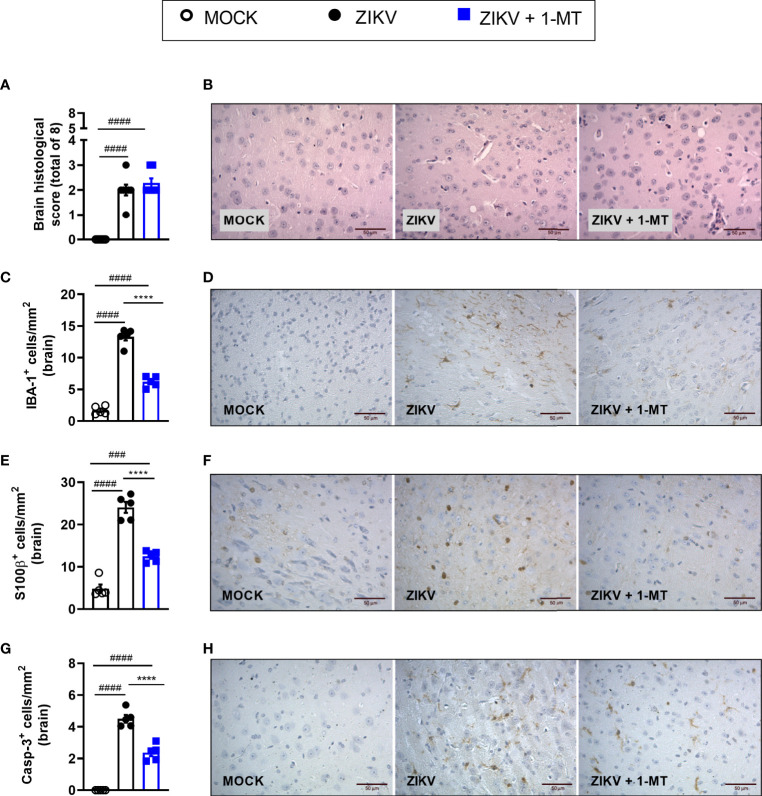
Morphometric analysis of the brain of A129 mice infected by ZIKV, treated or not with 1-MT inhibitor. A129 were inoculated (i.v) with 4x10^3^ PFU/200μL of ZIKV and treated daily with 1-MT. **(A)** Semiquantitative analysis (histopathological score) after H&E staining of brain sections of ZIKV-infected mice 5 days after infection. **(C, E, G)** Immunostaining of **(C)** IBA-1^+^, **(E)** S100-β^+^ and **(G)** Caspase-3 was performed in the brains of mice. **(B, D, F, H)** Representative images from brain sections. All results are expressed as mean and error bar indicate the standard error (SEM) and are representative of at least two independent experiments. Statistically significant differences were assessed by One-way ANOVA plus Tukey’s comparisons test. (###) for *P < 0,001* and (####) for *P < 0,0001* when compared to the MOCK group. (****) for *P < 0,0001* compared to the ZIKV group.

Infection of A129 mice with ZIKV triggered histopathological signs of brain inflammation and tissue damage. Histopathological analysis of the brain revealed mild gliosis and meningitis 5 days after ZIKV infection. Such changes were similar in non-treated and 1-MT-treated ZIKV-infected mice (**Figures 4A, B**). Despite such evidence, 1-MT treatment prompted a significant reduction in the number of IBA-1 positive cells (IBA-1^+^ cells) in the cerebral cortex of infected mice (**Figures 4C, D**). In addition, there was significant reduction in the number of astrocytes (S100β^+^ cells) in 1-MT-treated group when compared to non-treated ZIKV infected group (**Figures 4E, F**). Another important feature of ZIKV infection is neurodegeneration, including cell death. In this regard, treatment of infected mice with 1- MT decreased the number of brain Caspase-3-positive cells (Caspase-3^+^ cells), which is suggestive of reduced apoptosis (**Figures 4G, H**).

### Effects of ZIKV Infection in Indoleamine 2,3-Dioxigenase 1 (IDO-1^-/-^) Deficient Mice

**Figure 5:**
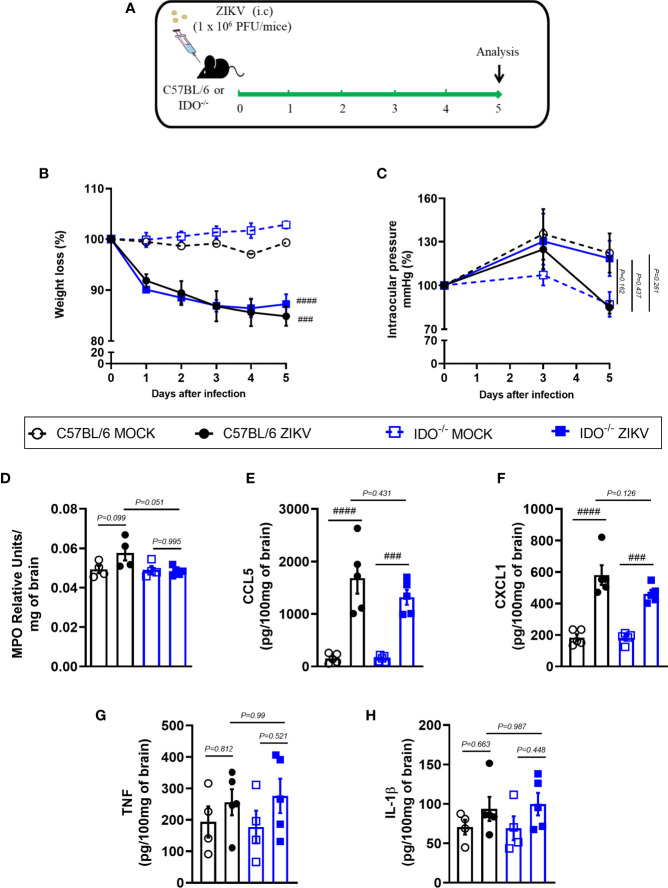
Clinical signs and inflammatory parameters after ZIKV infection of WT and IDO^-/-^ mice infected by ZIKV. **(A)** Experimental schematic representation of experimental strategy. C57BL/6 and IDO-1^-/-^ were inoculated (intracranial) with 1x10^6^ PFU/20μL of ZIKV. **(B)** The body weight was evaluated daily and **(C)** the intraocular pressure was analyzed on days 0, 3 and 5. At day 5 of infection, **(D)** the brain was harvested and myeloperoxidase absorbance analysis and **(E–H)** chemokine/cytokine production was assessed. All results are expressed as mean and error bar indicate the standard error (SEM) and are representative of at least two independent experiments. Statistically significant differences were assessed by Two-way ANOVA plus Tukey’s comparisons test **(B, C)** and One-way ANOVA plus Tukey’s comparisons test (D-H). (###) for *P < 0,001* and (####) for *P < 0,0001* compared to the MOCK group.

**Figure 6:**
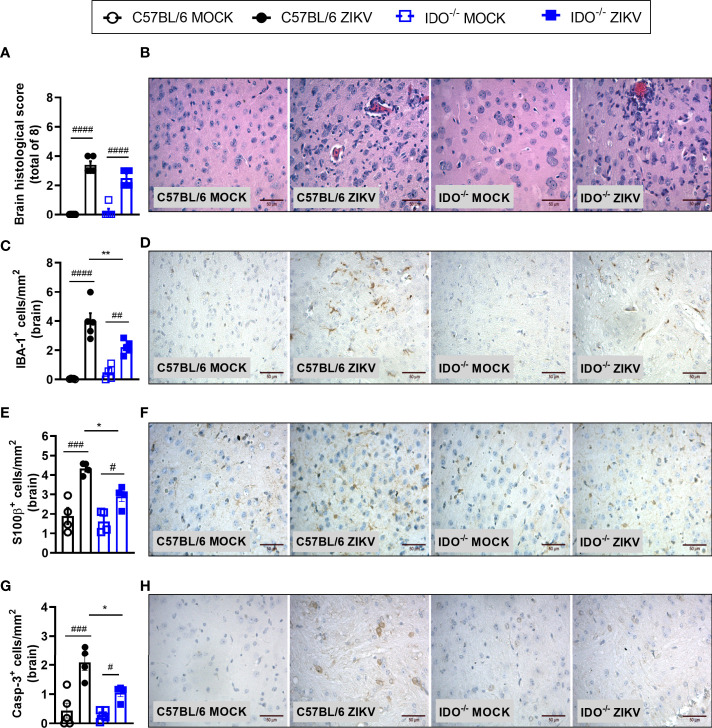
Morphometric analysis of the brain of WT and IDO^-/-^ mice infected by ZIKV. C57BL/6 and IDO-1^-/-^ were inoculated (intracranial) with 1x10^6^ PFU/20μL of ZIKV. **(A)** Semiquantitative analysis (histopathological score) after H&E staining of brain sections of ZIKV-infected mice 5 days after infection. **(C, E, G)** Immunostaining of **(C)** IBA-1^+^, **(E)** S100-β^+^ and **(G)** Caspase-3 was performed in the brains of mice. **(B, D, F, H)** Representative images from brain sections. Results are expressed as mean and error bar indicate the standard error (SEM) and are representative of at least two independent experiments. Statistically significant differences were assessed by One-way ANOVA plus Tukey’s comparisons test (#) for *P < 0,05*, (##) for *P < 0,01* and (###) for *P < 0,001* and (####) for *P < 0,0001* when compared to the MOCK group. (*) for *P < 0,05* and (**) for *P < 0,01* compared to the ZIKV group.

In view of the effects observed after 1-MT treatment and the potential contribution of this pathway in preventing neurodegeneration, we also evaluated ZIKV infection in genetically modified Indoleamine 2,3-dioxigenase 1 deficient (IDO-1^-/-^) mice. Since C57BL/6 mice are resistant to ZIKV infection (27, 34, 35), ZIKV infection was performed by intracerebral route by inoculation of with 1 x 10^6^ PFU of ZIKV (i.c) in both WT C57BL/6 and IDO-1^-/-^ mice (**Figure 5A**). Body weight loss was significantly higher in C57BL/6 and IDO-1^-/-^ mice infected with ZIKV than in their respective controls (**Figure 5B**). Furthermore, there was no significant change in the IOP levels between WT and IDO-1^-/-^ ZIKV-infected groups (**Figure 5C**). Regarding viral replication, no viable virus (assessed by plaque assay) or ZIKV genome (RT-PCR) was detected in the brain of ZIKV-infected WT or IDO-1^-/-^ mice. Accordingly, no difference in MPO levels was detected into mice’s brains (**Figure 5D**). Finally, as demonstrated during 1-MT treatment, intracranial ZIKV infection also led to increased production of inflammatory mediators, such as CCL5 (**Figure 5E**) and CXCL1 (**Figure 5F**) in both WT and IDO-1^-/-^ ZIKV mice. Production of TNF and IL-1β in WT and IDO-1^-/-^ was analogous to Mock (**Figures 5G, H**). Additionally, in a pattern similar to that observed in A129 mice treated with 1-MT, brain histopathological analysis of C57BL/6 and IDO-1^-/-^ infected mice revealed mild gliosis and meningitis in both infected experimental groups (**Figures 6A, B**). Accordingly, reduced microgliosis, astrogliosis and apoptosis were found in infected IDO-1^-/-^ mice in comparison to ZIKV infected WT littermates as demonstrated by reduction of the number of IBA-1 (**Figures 6C, D**), S100β (**Figures 6E, F**) and Caspase-3 (**Figures 6G, H**) positive cells of mice’s brain sections. Furthermore, the morphology of the Caspase-3-stained cells, especially the nucleus morphology, comprised both neurons and glial cells.

## Discussion

Several studies have reported the tropism of the ZIKV to different cellular types, tissues and body fluids. Meanwhile, ZIKV-tropism to the CNS is the most harmful feature to the host and may cause neurodegeneration and other neuronal dysfunctions. Despite its association with neurological diseases, such as microcephaly, GBS and CZS, there are no current approved therapies for the treatment of this important pathogen (36). In order to develop effective therapies, a better understanding of the pathogenicity resulting from the virus-host interaction is required. Global analysis of gene expression shows that ZIKV infection prompts significant dysregulation of several host factors associated with neural development, immune response, cell death, among others (37, 38). In the present study, we evaluated the potential neuroprotective effect of the blockade of IDO-1 enzyme by using a genetically approach (IDO-1^-/-^ mice) or a pharmacological strategy (1-MT inhibitor), *in vitro* and *in vivo*. The main results were: (I) increased levels of IDO-1 expression after ZIKV infection; (II) reduction of neuronal death upon 1-MT treatment; (III); massive reduction of ZIKV-induced microgliosis, astrogliosis and apoptosis in IDO^-/-^ mice and after 1-MT administration in A129 mice. Overall, these data indicate a neuroprotective effect induced by IDO-1 inhibition after ZIKV infection *in vivo* and *in vitro*.

IDO-1 is the first rate-limiting of the KP responsible for converting tryptophan into several downstream kynurenine metabolites. This enzyme is expressed by many cell types, including macrophages, microglia, dendritic cells, astrocytes, fibroblasts, and epithelial cells (32) and is commonly induced by proinflammatory cytokines. In the current study, we showed that ZIKV infection increased levels of IDO-1 expression in mice’s brain and also in primary neuron culture obtained from embryonic brains of mice. These results demonstrate that Indoleamine-2,3-dioxygenase, or IDO-1, is activated by the inflammatory response induced by ZIKV. In this context, we next, evaluated the effects of the inhibition of IDO-1 on ZIKV infection *in vitro* and *in vivo* systems. We found that a pharmacological inhibition in primary neurons infected by ZIKV did not prevent viral replication but induced less cell death. Likewise, human monocyte-derived macrophages induce IDO-1 expression upon West Nile virus (WNV), *via* TNF and NF-KB signaling, but IDO was not essential for the control of WNV infection (39). Similarly, 1-MT treatment of ZIKV-infected mice did not interfere with the ability of ZIKV to replicate in the brain, optic nerve, eye or spleen tissues. Additionally, no significant difference in neutrophil recruitment was observed between treated and non-treated ZIKV-infected mice. At the same time, chemokines relevant for the migration of monocytes, lymphocytes, as well as for neutrophil traffic, such as CCL5 and CXCL1, respectively, had increased levels in 1-MT treated ZIKV mice. We also evaluated ZIKV infection in IDO-1^-/-^ mice and WT mouse model. Corroborating the pharmacological data, the absence of IDO did not interfere with clinical signs, viral replication or inflammatory parameters, although microgliosis, astrogliosis and caspase-3 expression reduced after infection. Chemokine signaling plays an important homeostatic role and modulates neuroprotective processes on different stimuli, orchestrating the action of neurons-microglia-astrocytes to preserve brain damage (40). In pathological processes, pro-inflammatory cytokines and chemokines are robustly induced and can lead IDO-1 activity and tryptophan associated metabolites (39, 41, 42). IDO-1 inhibition in Influenza virus infection does not affect leukocyte infiltration or viral clearance, thus, blockade of IDO-1 could play a beneficial role in host protection (43–45). These data show that pharmacological inhibition or genetic ablation of IDO-1 did not completely prevent brain inflammation, but reduced brain damage and cell death.

ZIKV can infect almost all CNS cells, including neurons, glial cells, endothelial cells and pericytes. Activated CNS-resident cells could be the source of proinflammatory cytokines and chemokines such as CCL5 and CXCL1, found in ZIKV-infected brain tissue. ZIKV infection and replication are associated with brain damage and, as consequence, severe neurological symptoms (8, 27). The mechanisms by which ZIKV exert these effects involve neuronal excitotoxicity and apoptosis of microglia, astrocytes and other cells. Our group has shown that neuronal cell death induced by ZIKV is associated with excitotoxicity mediated by the release of glutamate and other neurotoxic factors such as TNF and IL-1β, inducing the activation of NMDA (N-methyl-d-aspartate receptor) receptors (27, 33). Accordingly, treatment with Memantine (an FDA-approved allosteric NMDA receptor antagonist) *in vivo* and *in vitro* was effective to prevent ZIKV-induced cell death and neurodegeneration but did not interfere with the ability of the virus to replicate. Furthermore, administration of Ifenprodil (a selective inhibitor of the NMDA receptor, specifically for the GluN1 and GluN2B subunits) in a primary neuron culture rescues ZIKV-induced neurotoxicity (27, 33). IDO expression in Huntington’s disease is chronically elevated, inducing neurotoxicity, and IDO inhibition reduces neurotoxicity sensitivity and neuroprotective role (46).

Cell death and neurodegenerative processes can be exacerbated by metabolites produced by KP. Kynurenine metabolism is directly associated with several neurodegenerative and neurological diseases, such as Alzheimer’s disease, multiple sclerosis, and stroke (23, 47). In accordance with recent studies from our group (27, 33, 48), infection of A129 mice induced brain inflammation and tissue damage, and we found mild gliosis and meningitis in all infected mice groups. However, pharmacological inhibition or deletion of IDO-1 (IDO^-/-^) prompted a significant reduction of IBA-1 positive cells in the brain’s mice, suggesting less microglia activation. It appears that microglia activation is dependent on IDO-1 (49, 50). In a similar pattern, we found a reduction in the number of astrocytes (S100β positive cells). Microglia and activated astrocytes produce several inflammatory mediators, including IL-1β, TNF, glutamate, nitric oxide (NO) among others, which can trigger neuronal death after ZIKV infection (33). In addition, ZIKV-induced neurodegeneration can be caused by loss of structure or function of neurons. Recent studies demonstrated that ZIKV is able to induce apoptosis of neuronal cells *in vitro* (33) and *in vivo* (28). In this regard, we have shown that treatment of infected mice with 1-MT decreased the number of brain Caspase-3-positive cells, which is suggestive of reduced apoptosis. According to cell morphology, especially the nucleus, these cells comprised both neurons and glial cells, however, co-localization assays were not performed (Caspase-3/IBA-1 or Caspase-3/S100β or NeuN/Caspase-3). Although ZIKV-induces neuronal damage, it is unclear whether neuronal death is due to direct neurotoxicity or secondary to the activation of microglia and astrocytes. Apoptosis can be a result from inflammatory mediators released by the activated microglia or induced in a non-cell autonomous manner, triggering cell death of uninfected neurons by releasing cytotoxic factors (33). These data suggest the pharmacological or genetic inhibition of IDO-1 prevents microglial activation, astrogliosis and apoptosis in the brain of ZIKV infected, without preventing clinical outcomes.

Development of efficient therapies against viral infections, such as ZIKV, is important for the control and reduction of clinical symptoms, viral load, neuroinflammation, and mainly, neurodegeneration. A combination of compounds that could prevent ZIKV-induced neurodegeneration with viral replication (antiviral drugs) represents the ideal treatment. Meanwhile, no approved antiviral drugs are available. Recently, our group showed that a therapeutic administration of an antiviral peptide (AH-D) significantly reduced viral loads in serum, spleen, brain and optical nerve throughout the course of ZIKV infection (30). Additionally, we also demonstrated the efficacy of the 7-Deaza-7-fluoro-2’-C-methyl adenosine (DFMA), a nucleoside analogue, which showed significant antiviral effects against ZIKV *in vitro* and *in vivo* systems, especially when administered prophylactically and at early times after ZIKV infection (48). These compounds open a new perspective to evaluate the synergic effect of a neuroprotective drug such as 1-MT along with an antiviral drug against ZIKV-induced disease in an early future.

In conclusion, our data indicate that IDO-1 inhibition exerts a neuroprotective role in CNS reducing the microglial activation, astrogliosis and apoptosis without antiviral effect. Further studies are required to elucidate the mechanisms of neuroprotection obtained by ablation of IDO-1 and it will be important to evaluate whether the combination of neuroprotective drugs, such 1-MT, and antiviral like AH-D or DFMA could be the treatment for ZIKV infection.

## Data Availability Statement

The raw data supporting the conclusions of this article will be made available by the authors, without undue reservation.

## Ethics Statement

The animal study was reviewed and approved by Committee on Animal Ethics of Universidade Federal de Minas Gerais (UFMG) (protocol no.106/2020 CEUA/UFMG).

## Author Contributions

FM, JA-F, TC, MT, AT, and VC designed the study. FM, DT, CQ-J, and BV conducted the experiments and analysis of the data. FM, AT, and VC interpreted the results. FM, CQ-J, AT, and VC wrote the first draft of the paper. RD, MT, AT, and VC obtained funding. All authors contributed to the article and approved the submitted version.

## Funding

This work was supported by the Grants from National Institute of Science and Technology in Dengue and Host-microorganism Interaction (INCT dengue), which is a programme sponsored by the Brazilian National Science Council (CNPq, Brazil) and the Minas Gerais Foundation for Science (FAPEMIG, Brazil). This work also received support from Coordenação de Aperfeiçoamento de Pessoal de Nível Superior – CAPES/Brazil (grant no CAPES: 88881.130741), ZIKALLIANCE consortium – 734548, Financiadora de Estudos e Pesquisa (FINEP 01.16.0050.00, Brazil), Conselho Nacional de Desenvolvimento Cientifico e Tecnológico (CNPq) under grants: 425359/2018 and 440423/2016-13, Fundação de Amparo à Pesquisa do Estado de Minas Gerais (FAPEMIG, Brazil) - APQ-02281- 18) and by L’Oréal-Unesco-ABC For Women in Science Program). Also, this work was funded by NIH (R01 CA193522 and R01 NS073939) and an MD Anderson Cancer Support Grant (P30 CA016672).

## Conflict of Interest

RD has done consultancy work for Compass Pathways, UK.

The remaining authors declare that the research was conducted in the absence of any commercial or financial relationships that could be construed as a potential conflict of interest.

## Supplementary Material

Supplementary Figure 1 Viral load in the eye and spleen of A129 mice infected by ZIKV, treated or not with 1-MT inhibitor. A129 were inoculated (i.v) with 4x10^3^ PFU/200μL of ZIKV and treated daily with 1-MT. **(A)** Eye and **(B)** spleen was harvested for plaque assay analysis. All results are expressed as mean and error bar indicate the standard error (SEM) and are representative of at least two independent experiments. Statistically significant differences were assessed by One-way ANOVA plus Tukey’s comparisons test.
