# Supplementary figures and images for: Inhibition of Tryptophan Catabolism Is Associated With Neuroprotection During Zika Virus Infection
Source: Front Immunol. 2021 Jul 15;12:702048. doi: 10.3389/fimmu.2021.702048 (PMC8320694; doi:10.3389/fimmu.2021.702048)

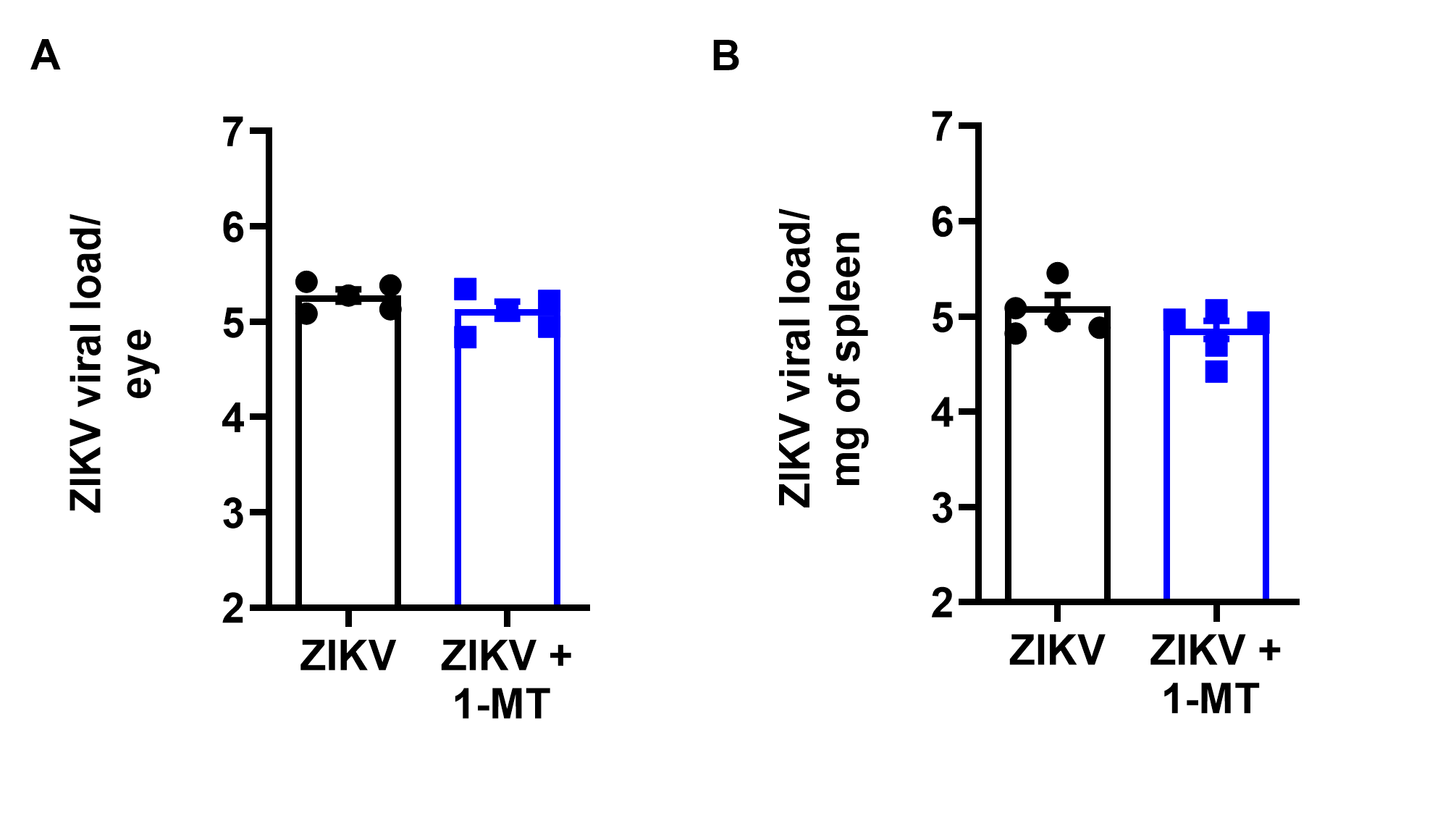

Supplement: Supplementary Figure 1 — Viral load in the eye and spleen of A129 mice infected by ZIKV, treated or not with 1-MT inhibitor. A129 were inoculated (i.v) with 4x10^3^ PFU/200μL of ZIKV and treated daily with 1-MT. (A) Eye and (B) spleen was harvested for plaque assay analysis. All results are expressed as mean and error bar indicate the standard error (SEM) and are representative of at least two independent experiments. Statistically significant differences were assessed by One-way ANOVA plus Tukey’s comparisons test. [file Image_1.tif]
